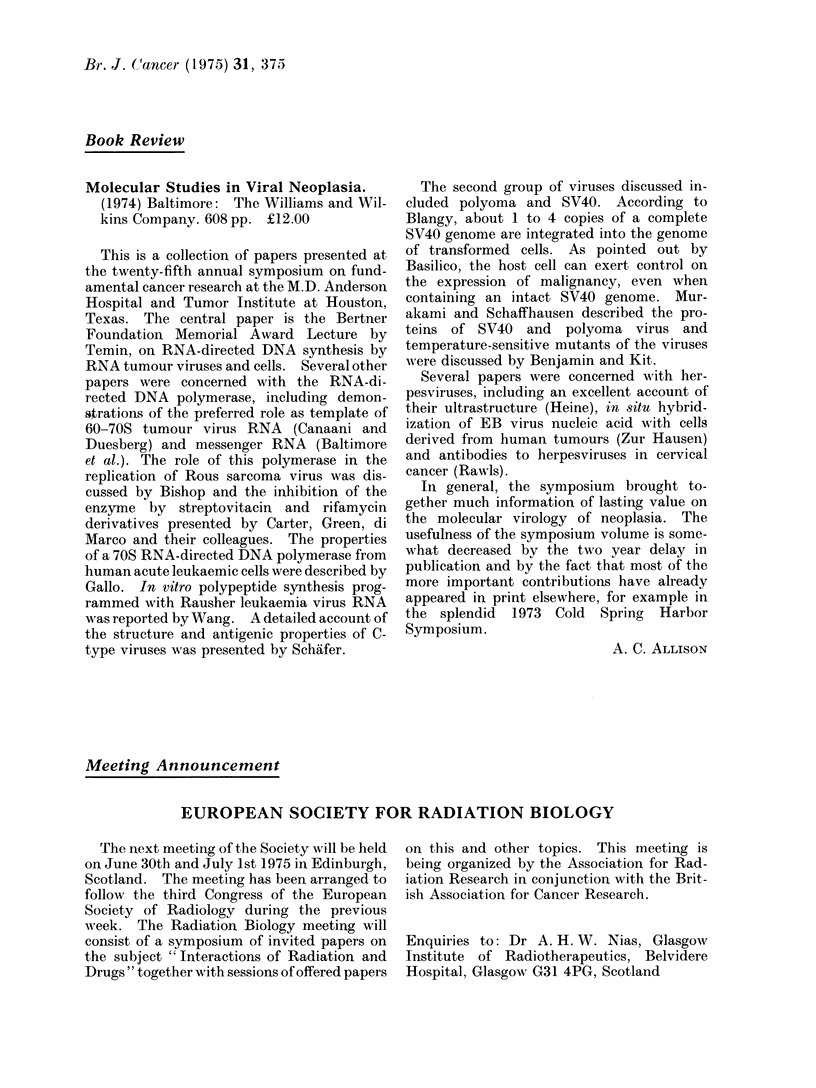# Molecular Studies in Viral Neoplasia

**Published:** 1975-03

**Authors:** A. C. Allison


*Br. J. Cancer* (1975) **31**, 375

### *Book Review*

**Molecular Studies in Viral Neoplasia.** (1974) Baltimore: The Williams and Wilkins Company. 608 pp. £12.00

This is a collection of papers presented at the twenty-fifth annual symposium on fundamental cancer research at the M.D. Anderson Hospital and Tumor Institute at Houston, Texas. The central paper is the Bertner Foundation Memorial Award Lecture by Temin, on RNA-directed DNA synthesis by RNA tumour viruses and cells. Several other papers were concerned with the RNA-directed DNA polymerase, including demonstrations of the preferred role as template of 60–70S tumour virus RNA (Canaani and Duesberg) and messenger RNA (Baltimore *et al.*). The role of this polymerase in the replication of Rous sarcoma virus was discussed by Bishop and the inhibition of the enzyme by streptovitacin and rifamycin derivatives presented by Carter, Green, di Marco and their colleagues. The properties of a 70S RNA-directed DNA polymerase from human acute leukaemic cells were described by Gallo. *In vitro* polypeptide synthesis programmed with Rausher leukaemia virus RNA was reported by Wang. A detailed account of the structure and antigenic properties of C-type viruses was presented by Schäfer.

The second group of viruses discussed included polyoma and SV40. According to Blangy, about 1 to 4 copies of a complete SV40 genome are integrated into the genome of transformed cells. As pointed out by Basilico, the host cell can exert control on the expression of malignancy, even when containing an intact SV40 genome. Murakami and Schaffhausen described the proteins of SV40 and polyoma virus and temperature-sensitive mutants of the viruses were discussed by Benjamin and Kit.

Several papers were concerned with herpesviruses, including an excellent account of their ultrastructure (Heine), *in situ* hybridization of EB virus nucleic acid with cells derived from human tumours (Zur Hausen) and antibodies to herpesviruses in cervical cancer (Rawls).

In general, the symposium brought together much information of lasting value on the molecular virology of neoplasia. The usefulness of the symposium volume is somewhat decreased by the two year delay in publication and by the fact that most of the more important contributions have already appeared in print elsewhere, for example in the splendid 1973 Cold Spring Harbor Symposium.

A. C. Allison